# Molecular targets for anticancer therapies in companion animals and humans: what can we learn from each other?

**DOI:** 10.7150/thno.55760

**Published:** 2021-02-06

**Authors:** Irati Beltrán Hernández, Jannes Z. Kromhout, Erik Teske, Wim E. Hennink, Sebastiaan A. van Nimwegen, Sabrina Oliveira

**Affiliations:** 1 Pharmaceutics, Department of Pharmaceutical Sciences, Faculty of Science, Utrecht University, 3584 CG Utrecht, the Netherlands.; 2 Cell Biology, Neurobiology and Biophysics, Department of Biology, Faculty of Science, Utrecht University, 3584 CH Utrecht, the Netherlands.; 3 Department of Clinical Sciences, Faculty of Veterinary Medicine, Utrecht University, 3584 CM Utrecht, the Netherlands.

**Keywords:** targeted therapy, comparative oncology, companion animals, molecular targets

## Abstract

Despite clinical successes in the treatment of some early stage cancers, it is undeniable that novel and innovative approaches are needed to aid in the fight against cancer. Targeted therapies offer the desirable feature of tumor specificity while sparing healthy tissues, thereby minimizing side effects. However, the success rate of translation of these therapies from the preclinical setting to the clinic is dramatically low, highlighting an important point of necessary improvement in the drug development process in the oncology field. The practice of a comparative oncology approach can address some of the current issues, by introducing companion animals with spontaneous tumors in the linear drug development programs. In this way, animals from the veterinary clinic get access to novel/innovative therapies, otherwise inaccessible, while generating robust data to aid therapy refinement and increase translational success. In this review, we present an overview of targetable membrane proteins expressed in the most well-characterized canine and feline solid cancers, greatly resembling the counterpart human malignancies. We identified particular areas in which a closer collaboration between the human and veterinary clinic would benefit both human and veterinary patients. Considerations and challenges to implement comparative oncology in the development of anticancer targeted therapies are also discussed.

## Introduction

Cancer is an important cause of death in humans worldwide, accountable for approximately 10 million deaths every year 1. The use of surgery, chemo- and radiotherapy has certainly led to remarkable clinical outcomes for some early stage cancers 2, but the overall need for improved outcome is undeniable. Differences in anatomical location, aetiology, and molecular biology of cancers stress the necessity to develop novel therapies and combined approaches that more specifically address the cancer in an individual patient. Expanding the therapeutic arsenal to fight cancer, numerous targeted therapies and immunotherapies have emerged in the last decade 3, 4. In particular, continuous development in the field of molecular biology and oncogenes raises opportunities for new molecular targeted therapies, e.g. tyrosine kinase inhibitors (TKI), monoclonal antibodies and antibody-drug conjugates targeting cancer cells. These offer selectivity towards cancer cells over normal cells by exploiting genetic alterations in malignant cells, minimizing damage to healthy tissues and improving patient's quality of life 4. This attractive feature unique to targeted therapies warrants the innovative efforts of researchers to develop new targeted treatments.

In the oncology field, the success rate of translation of a therapy from preclinical to clinical use is as low as 3.4% 5, 6. This is the lowest success rate among therapeutic disciplines and can be partly explained by the use of (immunocompromised) rodent xenograft models as the main experimental tool in the field, which certainly fail to reproduce most aspects of such an intricate disease as cancer. These models, in addition to the obvious biological differences between species, often miss the tissue-specific environment, host immunity, the capacity to metastasize and development of treatment resistance 6. It is clear that a more clinically relevant setting is needed to bridge the currently existing gap and lead to more successful therapies. Comparative oncology offers this, being a discipline that integrates the study of spontaneous cancers in companion animals into studies of human oncology. Consequently, a win-win scenario is created in which therapeutic options can arise for both humans and veterinary patients. This field of study exemplifies the concept of “One Medicine”, which recognises the unity of human and veterinary medicine and that both can advance hand in hand 7. Dogs and cats with naturally occurring tumors and intact immune systems are of extraordinary translational significance 8. These veterinary patients present a larger genetic variation than inbred mouse strains and are exposed to environmental factors similar to humans, resulting in less clear-cut results during clinical evaluation that better reflect the human situation. In addition, the shorter life span and lack of therapeutic options for companion animals allow the smooth initiation of therapeutic trials and rapid data collection, while still offering a relatively long-term follow-up 8. Many cancer types in the veterinary clinic are considered as exceptional models of the human counterpart, exhibiting genetic, molecular and clinical similarities 9, 10. With the revealing of the dog genome in 2005 and, more recently, the cat genome in 2014, the study of spontaneous cancers in these animals has become even more interesting for comparative oncology 11, 12.

**Figure 1:**
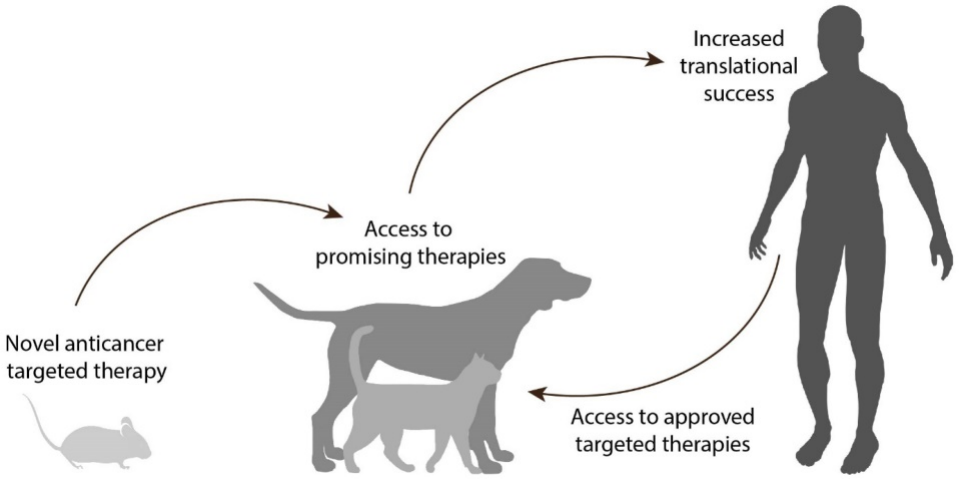
** Proposed approach for the development of anticancer targeted therapies.** First, encouraging results are obtained in preclinical studies (e.g. rodents) for a targeted therapy utilizing a particular target. The therapy can be evaluated for the treatment of relevant cancers in companion animals expressing the target in question, allowing access to novel and promising therapeutic approaches for these veterinary patients. Robust data are thus acquired in a more relevant setting, to guide the translation of the therapy towards the equivalent human cancer while increasing translational success. At the same time, the use of current successful targeted therapies in the human clinic can be expanded to the veterinary clinic, based on the knowledge of which targets are present in the different canine and feline cancers.

In the context of developing novel targeted therapies, the treatment of veterinary patients with spontaneous tumors can generate robust data and give very valuable insights for treatment refinement, thereby increasing the translational success rate. At the same time, these animals get access to novel and promising therapies, which would otherwise remain inaccessible for these patients (Figure 1).

In this review, we present an overview of molecular targets in companion animals and their expression in relevant cancers (i.e. with translation potential). The focus is on targetable membrane proteins with high homology across species present on the most well-characterized solid cancer models: canine and feline oral squamous cell carcinoma, canine bladder cancer, canine and feline mammary carcinoma, canine osteosarcoma and canine melanoma. A brief introduction of each cancer, its human counterpart and available targeted therapies is given in Table 1, together with a collection of comparative studies highlighting the rationale to focus on the selected cancer types. This table evidences the existing gap between human and veterinary medicine, since no targeted therapies are yet approved for the reviewed cancer types in companion animals. From the listed targeted therapies in Table 1, only the approved monoclonal antibodies cetuximab and trastuzumab are being assessed at the preclinical level for the treatment of the veterinary malignancies, and thus these therapies will be addressed further in this review.

The different membrane targets that are addressed in this review are presented in Table 2, emphasizing their relevance in the oncology field. Most of these proteins are tyrosine kinase receptors mediating cell proliferation and survival. G protein-coupled receptors and nutrient transporters are also common targets under evaluation in human clinical trials. Table 2 lists some examples of the therapies approved for human use, mostly TKIs (e.g. gefitinib and lapatinib) and the already mentioned monoclonal antibodies (i.e. cetuximab and trastuzumab). Next to these, also other therapies are indicated in Table 2, though only those that were found to be tested in companion animals are described further.

Knowing the targets overexpressed in these relevant animal cancers, not only serves to guide the evaluation of targeted therapies in clinically relevant models of the human disease, but also to expand the use of current successful targeted therapies in humans towards the veterinary clinic. The feasibility of the proposed approach is supported by the fact that cancer remains a leading cause of death not only in humans, but also in companion animals. To compare, dogs develop cancer at a similar rate as humans (1 in 3), while cats are less affected (1 in 4 to 5) 26. About 50 % of dogs and 30 % of cats over age ten die from cancer. Most of the discussed animal cancers are often compared to adult human cancers, since those are rare during childhood and have a higher incidence in geriatric animals, while canine osteosarcoma is considered to closely resemble the pediatric cancer 27.

## Epidermal growth factor receptor

### Head and neck squamous cell carcinoma

EGFR overexpression has been reported in up to 90% of human head and neck squamous cell carcinomas (HNSCC) and also in feline oral squamous cell carcinoma (OSCC), making it an interesting therapeutic target in both species 39-41. Although several EGFR-targeting small molecule inhibitors and monoclonal antibodies are currently approved and under clinical evaluation for the treatment of human HNSCC 42, 43, clinical evaluation of agents targeting EGFR in feline OSCC has not been reported. *In vitro* studies with a feline OSCC cell line have been conducted to target EGFR with the TKI gefitinib and RNA interference 44, 45. While gefitinib caused a reduction in cell proliferation, this was accomplished at a relatively high dose and gefitinib resistance developed. By combining siRNA with gefitinib treatment, it was possible to surpass the acquired resistance. Interestingly, the combination of siRNA targeting of EGFR and radiation therapy showed an additive cytotoxic effect in the feline cell line 44. These results, although scarce and in an initial stage, support the notion of EGFR targeting to treat feline OSCC.

### Invasive transitional cell carcinoma

EGFR is overexpressed in both human and canine invasive transitional cell carcinoma (TCC) in about 75% of the cases 17, 46, 47. Several EGFR-targeting compounds have been or are currently evaluated in clinical trials of human TCC, but so far with mixed results 17. Recently, a particular 67-gene signature has been associated with sensitivity to EGFR inhibition in human bladder cancer cell lines 48. Regarding canine invasive TCC, there is only one clinical study involving EGFR-targeting as therapy 49. In this study, an EGF‐conjugated anthrax toxin was used to target EGFR-expressing human and canine TCC cell lines. After encouraging *in vitro* results, the effects of the EGF-toxin were evaluated in 6 dogs with invasive TCC, resulting in a 30% reduction of the tumor size in all dogs. These data show promising effects and follow-up studies are warranted. In another recent study, near-infrared photoimmunotherapy was used to target EGFR in canine invasive TCC cell lines 50. The EGFR-targeting monoclonal antibody can225IgG (caninized cetuximab) was conjugated to a photosensitizer (IRDye700DX) and tested in canine TCC cell lines, showing specificity and cytotoxic potency. Using a xenograft mice model of canine invasive TCC, NIT-PIT treatment led to a statistically significant inhibition of tumor growth and prolonged survival, highlighting thus the promise of the approach.

### Osteosarcoma

EGFR is frequently overexpressed in human osteosarcoma (OSA); however, the exact contribution of EGFR in OSA prognosis and development is still not fully understood 51, 52. EGFR is expressed in canine OSA cell lines and tissues, and a higher expression correlates with a poor prognosis 53, 54. Currently, several ongoing clinical trials evaluate the use of EGFR-targeting agents in human patients with OSA 52. As for canine OSA, only *in vitro* data are available so far 54-56. Three small molecule EGFR-targeting TKIs, i.e. erbstatin analogue, erlotinib and gefitinib, have been tested alone or in combination with different therapeutic approaches. The three drugs showed activity as monotherapy, but combinations yielded better results. In particular, erbstatin analogue was tested in combination with the chemotherapy agent doxorubicin, resulting in increased cell death compared to erbstatin monotherapy. Erlotinib was tested in combination with radiation therapy and enhanced sensitivity to radiation was observed. Lastly, the combination of gefitinib and the c-Met inhibitor crizotinib showed an additive effect on cell proliferation inhibition. Altogether, combination strategies involving EGFR targeting show potential for the treatment of canine OSA.

### Mammary carcinoma

EGFR expression is found in 15-45% of human breast cancer tissues 57. Accordingly, the EGFR TKIs gefitinib, erlotinib, lapatinib and afatinib are approved for the treatment of some subsets of breast cancer, while the evaluation of more EGFR-targeting drugs is ongoing in clinical trials 58. In canine mammary carcinoma (CMC) tissues, similar EGFR expression as in the human equivalent cancer has been reported 59. Similarly, EGFR was found to be significantly overexpressed in cell lines, tissues and xenograft mouse models of feline mammary carcinoma (FMC) 60. Several studies report cell proliferation inhibition induced by EGFR-targeting in CMC cell lines. Cetuximab and its caninized version (can225IgG), gefitinib and EGFR siRNA have all inhibited cell proliferation as a single agent 61-63*.* Only in one of these studies a FMC cell line was included, also showing cell proliferation inhibition after gefitinib treatment 62.

### Melanoma

EGFR has also been of interest as a therapeutic target in human melanomas, and it can play a role in the progression of cutaneous melanomas 64. Two clinical phase II trials have been conducted with EGFR-targeting agents, but the outcomes were poor in human patients with metastatic melanomas 65, 66. Therefore, there are currently no EGFR-targeted therapies approved for human melanomas. In canine melanoma, little is known regarding EGFR expression and its role. One recent study reported the expression of EGFR and other receptors from the EGFR family in canine melanomas, concluding that EGFR overexpression occurs only in 5% of the oral melanomas and 16% of the skin melanomas 67. In addition, an increased proliferative index was associated with a decreased EGFR expression in the skin melanomas. In another recent study, the expression of EGFR was found downregulated in oral melanoma compared to the healthy oral tissue 68. Therefore, EGFR does not seem to be a promising target in human and canine melanoma, but targeting of this receptor in the canine disease has not been investigated yet.

## Human epidermal growth factor receptor 2

### Invasive transitional cell carcinoma

HER2 overexpression is common in human TCC and it is, therefore, an interesting target 69. Likewise, significant overexpression of HER2 compared to normal canine bladder has been described in canine invasive TCC tissues at both the RNA and protein level 70-72. While multiple clinical trials are ongoing to evaluate different HER2-targeting drugs in human patients with TCC 73, the targeting of this protein has not been yet studied in the context of canine TCC. The outcome of the current human clinical trials will certainly shed light on the value of targeting HER2 in this type of bladder cancer and may spur research also in the field of veterinary oncology.

### Osteosarcoma

The role of HER2 in human OSA has been controversial. Multiple studies report overexpression of HER2, while others describe the opposite 74-76. As for canine OSA, overexpression of HER2 has been detected in different OSA cell lines and tissues at the RNA level 77. Despite the controversial findings on HER2 in human OSA, a clinical trial has been conducted with trastuzumab, a HER2-targeting monoclonal antibody, in combination with chemotherapy 78. The outcome was rather disappointing and HER2 positive patients did not have a significant therapeutic benefit. Nevertheless, HER2 seems to remain an interesting target in these cancers for targeted therapy and, particularly, for immunotherapy 52. In human patients with HER2-positive OSA, HER2-specific chimeric antigen receptor T cell therapy has shown initial promising results 79. Furthermore, dogs with HER2-positive OSA were successfully treated with a vaccine consisting of recombinant Listeria bacteria expressing a chimeric human HER2, which induced HER2-specific immunity and reduced the incidence of metastasis 80. Altogether, it is reasonable to consider immunotherapies involving HER2 targeting as a therapeutic option for both human and canine OSA, but the targeting of this receptor alone has not yet shown promise.

### Mammary carcinoma

HER2 is a major marker for human breast cancer classification and its presence or absence mainly guides the choice of therapeutic strategy. HER2 overexpression is found in 10-40% of human breast cancers 81. For the treatment of HER2-positive breast cancer, multiple HER2-targeting agents are already approved, e.g. trastuzumab, pertuzumab and afatinib, and others are being tested in ongoing clinical trials 58. In CMC and FMC, HER2 overexpression is in the same range as in human breast cancers 82-85. Nonetheless, studies of HER2-targeting in these veterinary patients have been so far limited to *in vitro* evaluations. The anti-HER2 antibody trastuzumab was able to inhibit cell proliferation in two CMC cell lines 61. On the other hand, using other CMC and FMC cell lines, the use of the HER2 inhibitor AG825 or a HER2 siRNA only caused a slight inhibition of cell proliferation 62. All things considered, the genetic, molecular and clinical similarities between the human and canine/feline disease support the value of targeting HER2 in companion animals with mammary carcinoma.

### Melanoma

The role of HER2 in human melanomas is still under debate, because of contradictory results regarding the overexpression of this target 86-88. In canine oral and skin melanomas, the role of HER2 is still largely unknown and there is only one study addressing this. HER2 membranous expression was found in 50 and 43% of the oral and skin melanomas, respectively, but this was not considered to be overexpressed 67. HER2 expression in the oral melanomas was associated with tumor progression, because of its correlation with emboli occurrence. So far, no solid conclusion can be drawn regarding the potential of HER2 targeting to treat melanoma.

## Platelet-derived growth factor receptors

### Osteosarcoma

Both PDGFRα and PDGFRβ are frequently overexpressed in OSA human tissues 89, 90. In canine OSA tissue, the expression of both proteins is commonly found and, in canine OSA cell lines, overexpression of the two receptors has been reported at the RNA level 91. However, clinical trials evaluating different PDGFR-targeted therapies in human OSA have not been successful. As a result, it has been suggested to target PDGFRs not as monotherapy, but rather in combination with other therapeutic agents such as check point inhibitors or chemotherapy 90. There are no studies available in which the targeting of PDGFRs in canine OSA has been investigated, but combination strategies might be the answer as in the case of the human disease.

### Melanoma

In human cutaneous melanomas, overexpression of PDGFRα is associated with decreased cell proliferation, whereas the role of PDGFRβ in melanomas is, on the other hand, less clear 92, 93. Still, several phase II and III clinical trials are being conducted in patients with malignant melanomas evaluating TKIs that target PDGFRs amongst other tyrosine kinase receptors 94. In canine oral melanomas, the co-expression of PDGFRα and PDGFRβ has been described in 37.5% of the cases, which corresponded with a significant shorter overall survival compared to dogs without co-expression 95. Although the targeting of exclusively PDGFRs has not been investigated yet in companion animals with melanoma, the encouraging results from human clinical trials may instigate researchers in this direction.

## Hepatocyte growth factor receptor

### Osteosarcoma

c-Met is frequently found overexpressed in human and canine OSA tissues and cell lines 54, 96-98. Nevertheless, to date, there are no clinical data available of c-Met-targeting agents to treat OSA. The two approved multi-TKI cabozantinib and crizotinib, which target c-Met amongst others, have been tested in human OSA cell lines and xenograft mouse models with promising results 99, 100. Liao et al. tested a c-Met-targeting drug called PF2362376 in canine OSA cell lines, which inhibited c-Met and induced cell death at a high dose 101. Another drug that targets c-Met, called PHA‐665752, and a c-Met shRNA have also been evaluated as single agents in two canine OSA cell lines. Although the two agents did not affect cell proliferation, the motility of the cell lines was decreased 98. Although not clear yet whether targeting c-Met alone can be of value, there are indications that c-Met inhibition can be a promising strategy to treat OSA when also other oncogenic pathways are being targeted.

## Insulin-like growth factor-1 receptor

### Osteosarcoma

The role of IGF-1 and IGF-1R has been investigated in several human cancers, including OSA 32. A significant overexpression of IGF-1R has been found in human OSA tissues compared to healthy bone tissue 102. In canine OSA cell lines and tissues, high expression of IGF-1R is frequently observed at both the RNA and protein level 103, 104. One clinical study assessing the efficacy of an IGF-1R-targeting drug has shown poor results in humans with OSA 105. Nonetheless, more IGF-1R-targeting drugs are being investigated in clinical trials to treat human OSA 106, while no data are available concerning canine OSA. The results of the ongoing human clinical trials, when promising, may guide the use of similar therapeutic strategies in dogs with OSA.

### Mammary carcinoma

Expression of IGF-1R is frequently found in human breast cancer with varying percentages in the different cancer subtypes 107. Therefore, IGF-1R has been an interesting target in breast cancer and multiple clinical trials have taken place with IGF-1R-targeting agents 108. However, these clinical trials showed poor outcomes, resulting in no approved IGF-1R-targeting drugs in human breast cancer so far. In multiple studies with tissue samples of canine mammary gland carcinomas and invasive CMC, high expression of IGF-1R was commonly found, but contradictory findings have been reported regarding its correlation with prognosis in CMC 109-111. The role and expression of IGF-1R has not been studied in FMC, and no therapeutic strategies targeting this receptor have been assessed in CMC or FMC. Clearly, while human data discourages IGF-1R targeting in breast cancer, more research is needed to get a better understanding of the potential of this approach in companion animals with mammary carcinoma.

### Melanoma

IGF-1R often plays a role in the different types of human melanomas and, therefore, it has been considered an interesting drug target for this disease 112, 113. Among multiple tyrosine kinase receptors, IGF-1R has been found to be the most abundant in canine malignant melanoma cell lines, and its expression was subsequently confirmed in the majority of canine melanoma tissues 114. There are several preclinical studies showing promising effects of IGF-1R targeting in combination with other therapeutic agents for the treatment of human melanoma 115. Nevertheless, the use of IGF-1R-targeting agents has not been evaluated in canine melanomas, neither *in vitro* nor *in vivo*. It is reasonable to consider IGF-1R a good target in canine melanoma, based on its high expression and the so far encouraging results in human melanoma.

## Insulin-like growth factor-2 receptor

### Osteosarcoma

In contrast to IGF-1R, research conducted with IGF-2R as a therapeutic target is still in the initial phases. In human primary OSA cell lines, IGF-2R has been found to be significantly overexpressed 96. Monoclonal antibodies targeting IGF-2R conjugated to different cytotoxic radioisotopes, for radioimmunotherapy, have been evaluated in human OSA cell lines as well as patient-derived xenograft mouse models 116, 117. The authors showed specificity of the radiolabelled antibody and the ability to supress tumor growth. Furthermore, the same authors described IGF-2R expression in canine OSA tissue, making it an interesting target for further research 116.

## Folate receptor α

### Invasive transitional cell carcinoma

Dhawan et al. investigated the expression of FRs in human and canine invasive TCC tissues, which was found in 78 and 76% of the cases, respectively 118. In the same study, the FR-targeting drug folate-vinblastine conjugate was intravenously administered to treat 9 dogs with FR-positive invasive TCC. The drug led to partial remission in 56% of the dogs and stable disease in 44%. The same research group continued this line of investigation and treated 28 dogs with invasive TCC with a different FR-targeting drug, i.e. folate-tubulysin conjugate. This time, clinical benefit was observed in 71% of the dogs, including partial remission in 11% and stable disease in 60% 119. The encouraging results justify the assessment of FR-targeting agents to treat invasive TCC in the human clinic as well.

## Prostaglandin E2 receptor 2

### Invasive transitional cell carcinoma

The expression levels of EP2 in human urothelial cancer tissues have been shown to be significantly upregulated compared to normal urothelium 120. Interestingly, a decreased EP2 expression in the cytoplasm and nucleus in bladder cancer tissues compared to normal bladder has also been reported 121. When comparing the gene expression patterns of invasive canine TCC tissues and normal bladder, the gene encoding for EP2, PTGER2, was the most upregulated gene in the TCC samples 72. After that finding, EP2 protein expression was detected in the tumor cells of 11 out of 15 canine TCC tissues, whereas EP2 was absent in normal epithelial cells. Altogether, the overexpression of EP2 in human and canine TCC makes it a promising target, but the efficacy of targeting agents has yet to be investigated.

## Monocarboxylate transporters

### Head and neck squamous cell carcinoma

Increased expression of MCT1 and MCT4 in human oral SCC has been correlated with a poor survival prognosis and, therefore, MCT1 and MCT4 were proposed as therapeutic targets in HNSCC 122, 123. More recently, high expression of MCT1 in feline OSCC cell lines and tissues was reported, whereas MCT4 expression was relatively low 124. Accordingly, the action of a dual MCT1- and MCT4-inhibiting drug, MD-1, was tested in feline OSCC and human HNSCC cell lines as well as in an orthoptic feline OSCC xenograft mouse model. The targeting agent induced cell death in the human and feline cell lines and inhibited tumor growth in the orthoptic mouse model, highlighting the promise of MCTs as therapeutic targets in both species.

## CD146

### Melanoma

CD146 is highly expressed in human primary and metastatic melanomas and is a marker of poor prognosis in these patients 37. Multiple antibodies have been developed to target human CD146 and tested in preclinical settings with promising results 37, 125, 126. In canine tissues of oral and skin melanoma, CD146 is frequently highly expressed as well, especially in oral melanomas 127, 128. However, at present no studies have been published that investigated CD146 targeting in canine melanoma. Clinical trials to be conducted in the near future will give insights into the value of CD146 as a therapeutic target in the clinical setting.

## C-X-C chemokine receptor type 4

### Mammary carcinoma

Targeting CXCR4 in human breast cancer patients is currently under evaluation in clinical trials 129. In FMC tissues, CXCR4 is frequently highly expressed, whereas CXCR4 expression is not detected in healthy mammary tissues of cats 130, 131. Moreover, CXCR4 has been described as one of the top upstream regulators in canine mammary tumors 132. Further research in companion animals may be encouraged by the outcome of the current clinical studies in human patients 129.

### Multi-kinase inhibitors

Many targetable proteins are tyrosine kinase receptors on the cell membrane. Upon their activation (e.g. ligand binding), intracellular signaling occurs, inducing a multitude of cellular processes, including cell proliferation, differentiation and survival. Alteration in expression and/or activation of tyrosine kinase receptors is common in many human cancers and, therefore, these proteins have been of interest in the field of targeted therapeutics 133.

Sunitinib (Sutent®) and toceranib phosphate (Palladia®) are two drugs targeting multiple of these tyrosine kinase receptors simultaneously, including the vascular endothelial growth factor receptor (VEGFR), PDGFR, c-Kit, colony stimulating factor 1 receptor and fms-like tyrosine kinase 3 134-136. Sunitinib and toceranib phosphate are actually structural analogues, used for the treatment of cancer in the human and veterinary clinic, respectively. Sunitinib has been approved for the treatment of different cancers, such as renal cell carcinoma and gastrointestinal stromal tumors. Toceranib phosphate was the first drug approved for targeted cancer treatment in companion animals, indicated for canine mast cell tumors.

Another TKI with a broad range of targets is dasatinib (Sprycel, Dasanix), which targets several tyrosine kinase receptors such as c-Kit and PDGFRs, as well as the SRC kinase family of non-receptor signaling proteins. This drug has already been approved by the FDA for treatment of two types of human leukaemia 137, and its use in the veterinary clinic is gaining interest. Masitinib mesylate (Kinavet, Masivet) is another multi-TKI mainly inhibiting c-Kit and, to a lesser extent, other tyrosine kinase receptors such as PDGFRs and fibroblast growth factor receptor 3 (FGFR3) 138. Masitinib is approved for the treatment of canine mast cell tumors, while its evaluation in humans with solid tumors, such as pancreatic and colorectal cancer, is ongoing. An overview of the evaluation of toceranib phosphate, dasatinib and masitinib mesylate in the veterinary clinic (for the cancer models here reviewed) is shown in Table 3.

### Head and neck squamous cell carcinoma

The use of sunitinib has been evaluated in phase II clinical trials in recurrent or metastatic HNSCC, but resulted in poor outcomes due to low efficacy 146, 147. Since then, toceranib phosphate has been tested in different cancers in dogs and cats, including canine and feline OSCC 134, 135, 148. London et al. treated 8 dogs with carcinoma in the head and neck region with toceranib phosphate, and 6 of these dogs experienced clinical benefit 135. In a retrospective study of cats with OSCC, toceranib phosphate treatment significantly extended the lifespan compared to untreated cats 134. Here the treatment was combined with the administration of non-steroidal anti-inflammatory drugs (NSAIDs) and the beneficial effect of toceranib phosphate alone was not demonstrated. Nevertheless, overall, promising results have been observed so far for the use of toceranib phosphate in the treatment of OSCC in companion animals.

The use of masitinib, which has the highest affinity for c-Kit, has been evaluated in feline and canine OSCC cell lines. Overexpression and activation of c-Kit was found in human HNSCC 149, and similar expression levels have been detected in feline and canine OSCC cell lines 139. Masitinib inhibits the downstream pathway of c-Kit in these cell lines, but also induces expression of cyclooxygenase 2 (COX2), an enzyme involved in cell proliferation. Consequently, co-treatment with piroxicam (a COX2 inhibitor) resulted in the best inhibition of cell proliferation for the different SCC cell lines 139. Clearly, further research is still needed to elucidate the promise of these combination strategies.

### Invasive transitional cell carcinoma

Sunitinib has been evaluated in several phase II clinical trials of human patients with non-invasive or metastatic TCC. The general outcome of those studies was unsatisfactory, despite clinical benefit in some patients 150-152. The use of toceranib phosphate has been investigated for the treatment of canine TCC. The expression of PDGFRβ, VEGFR2 and c-Kit, all targets of toceranib phosphate, has been described in canine invasive TCC, although only PDGFR-β was found significantly overexpressed in comparison to normal bladder tissue 153. Owing to these findings, a retrospective study was recently performed regarding toceranib phosphate treatment in dogs with TCC, showing clinical benefit in 86.7% of the dogs 140. The study had some limitations, such as the co-administration of NSAIDs which can positively affect overall survival and the presumptive diagnosis of 12 out of 27 dogs. Nonetheless, the results are encouraging for the use of toceranib phosphate to treat canine TCC.

### Osteosarcoma

Sunitinib has shown promising effects in human OSA cell lines and xenograft mouse models 154, but no clinical data are available to date. The use of toceranib phosphate in canine OSA has been evaluated in the last couple of years. First, a retrospective study showed the first preliminary evidence of clinical benefits in canine OSA 135. However, follow-up studies failed to demonstrate a significant clinical benefit of toceranib phosphate as monotherapy or in combination with chemotherapy or other therapeutic agents 155-158. In a recent paper by Sánchez-Cespedes et al., toceranib phosphate was investigated in canine OSA cell lines and in two orthotopic xenograft canine OSA mouse models, resulting in decreased tumor growth only in one of the two mouse models 159. The OSA cell line used for this particular xenograft had high expression of PDGFR, c‐Kit and VEGFR2 and, as a result, it has been suggested that toceranib phosphate is more effective in tumors with high expression of these receptors.

Dasatinib has been tested in human clinical trials for the treatment of different sarcomas, including OSA, but there was insufficient activity as a single agent 160. In a single-case study of a dog with OSA, dasatinib was used as a treatment 141. The treated dog in the study first underwent surgery, by which primary tumor cells were obtained and cultured. Together with other canine OSA cell lines, the primary cells were screened for 86 small molecule inhibitors. Here dasatinib showed the most promising result and, consequently, was used to treat the dog after receiving surgery and chemotherapy. Eight months after completion of the treatment, the tumor had not recurred. In line with these observations, Marley et al. treated 4 dogs with OSA with dasatinib after surgery and chemotherapy, leading to a prolonged survival time and indicating the potential benefit of this drug for the treatment of canine OSA 142.

### Mammary carcinoma

Multiple phase III clinical trials of sunitinib treatment in patients with advanced breast cancer, either as monotherapy or in combination with chemotherapy, have not shown satisfactory results. Therefore, sunitinib has not been approved as treatment in human breast cancer 161-163. Evaluation of toceranib phosphate in a clinical setting to treat dogs bearing mammary carcinomas has been limited. Five dogs with mammary carcinoma were included and 2 of these had a partial response 144. Eighteen dogs with inflammatory mammary carcinoma were treated with toceranib phosphate in combination with piroxicam and thalidomide. Of those 18 dogs, 4 received additional radiation therapy, which led to partial response and significant increased survival time compared to the dogs which did not receive radiotherapy. For the 14 dogs not treated with radiation therapy, the combination treatment of toceranib phosphate, piroxicam and thalidomide caused a partial response in 3 dogs and 6 dogs experienced stable disease 143. Altogether, toceranib phosphate may be effective against canine mammary carcinoma in a multimodal approach, but the effectiveness as a single agent remains to be elucidated.

### Melanoma

Sunitinib has been evaluated in human clinical trials to treat cutaneous, mucosal and uveal melanomas. Nevertheless, sunitinib has shown limited activity in those trials and it has been suggested that sunitinib could be more beneficial in combination with other drugs for the treatment of melanoma 164, 165. In two canine clinical studies evaluating toceranib phosphate, a total of 4 dogs with melanoma were included, from which 3 dogs achieved a stable disease 144, 145. Although the first results are encouraging, the benefit of toceranib phosphate to treat canine melanomas needs to be addressed in larger studies.

The use of masitinib mesylate has also been evaluated in dogs with melanoma, strongly driven by the fact that c-Kit is a therapeutic target in human melanomas, especially in advanced melanomas with a mutated c-Kit gene 166. Different c-Kit inhibitors have already been tested in human clinical trials and imatinib is currently recommended for second-line treatment of c-Kit-mutated melanoma 167. In canine cutaneous and mucosal melanomas, only a few studies have investigated the expression, mutations and role of c-Kit, and the precise role of c-Kit is still not completely understood in these melanomas 168-171. In a recent study by Giuliano & Dobson, masitinib mesylate was evaluated as a treatment in 17 dogs with advanced malignant melanomas. Only 2 dogs with mucosal melanomas had a partial response, while 7 dogs achieved stable disease. The researchers concluded that masitinib mesylate treatment is not efficient, at least as monotherapy, for advanced malignant melanoma in dogs 172.

## Considerations for the development of targeted therapies in comparative oncology

**Figure 2:**
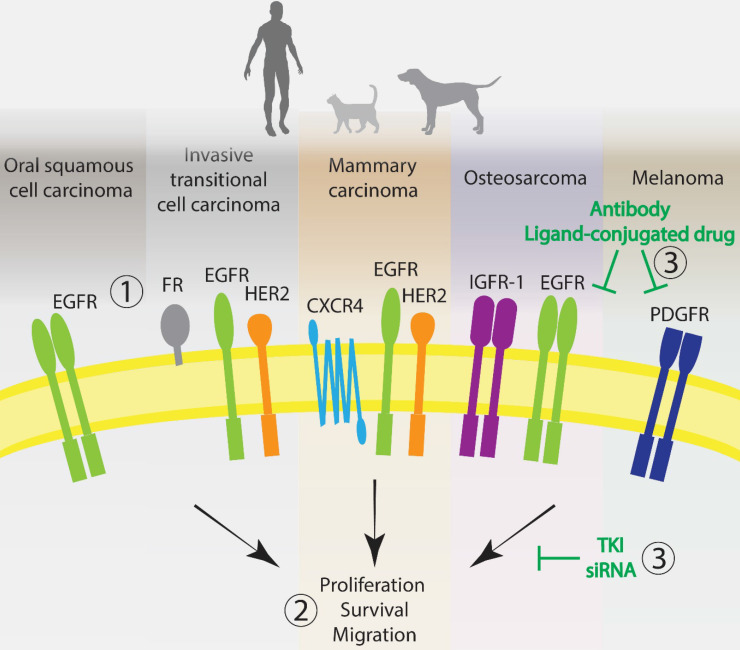
** Promising molecular targets for the development of anticancer targeted therapies in humans and companion animals.** 1. Most advanced and utilized targets with high homology across species for the five most well-characterized cancer types: EGFR and HER2. Other very promising targets: FR, CXCR4, IGFR-1 and PDGFR. 2. Signal deregulation of these membrane proteins, mostly tyrosine kinase receptors, leads to cell proliferation, survival, migration and tumorigenesis. 3. Categories of therapies most often used to interfere with the targets extracellularly or intracellularly. EGFR: epidermal growth factor receptor; HER2: human epidermal growth factor receptor 2; PDGFR: platelet derived growth factor receptor; IGF-1R: insulin-like growth factor 1 receptor; FR: folate receptor; CXCR4: C-X-C chemokine receptor type 4. TKI: tyrosine kinase inhibitor.

The use of targeted therapies to treat human cancers has been significantly more implemented than in the veterinary clinic 173. Nevertheless, with the latest developments in molecular biology and the veterinary approval of the two multi-TKIs toceranib and masitinib, targeted therapies in veterinary oncology are certainly becoming a more important focus of cancer research. Dogs with spontaneous cancers have been more of interest in comparative oncology than their feline comrades 10, 174. However, for some cancer types, cats can be more relevant for translational research; for instance, as a model for human HNSCC and metastatic breast cancer 10. Most advances in targeted therapies in the veterinary landscape are directed towards canine lymphomas and mast cell tumors. In this review, we have focused on the translation of targeted therapies for solid tumors, by presenting an overview of molecular targets in human cancers and the counterpart malignancy in companion animals, as well as the outcomes of the so far evaluated therapies. To enable relevant and translational research, we focused on targetable membrane proteins with high homology degree between species and their presence in five well-characterized solid cancer types, all considered exceptional models of the respective human disease. Table 4 presents a summary of the reviewed targets, their homology between human and dog or cat, the targeted therapies, and the stage of testing (i.e. cell lines, xenografts in mice, or companion animals). While multi-TKIs are more commonly evaluated in veterinary patients, the targeting of individual proteins is mainly restricted to preclinical research. So far, only the targeting of EGFR and FRα has been assessed in the clinic, to treat dogs with invasive TCC. A schematic representation of the most promising and advanced targets in the five different cancer types is depicted in Figure 2.

As evidenced by this review, EGFR-targeting receives large attention not only in human oncology, but also in veterinary research. Canine and feline EGFR share high homology with the human receptor (92%), making it an interesting target for translational research. In many human cancers, EGFR has proven an invaluable therapeutic target 28. Multiple EGFR-targeting monoclonal antibodies (e.g. cetuximab and panitumumab) and small molecule inhibitors (e.g. gefitinib and erlotinib) have been approved for the treatment of different human cancers, including HNSCC, colorectal and lung cancer 28, 43. In this regard, agents targeting EGFR have also been extensively investigated in canine and feline cell lines of OSCC, OSA, TCC and mammary carcinoma, but only the use of EGF‐anthrax toxin has been reported in the clinical setting, for the treatment of canine invasive TCC. This illustrates the large gap between the human and veterinary clinic and points at EGFR targeting as an area where closer collaboration between the human and veterinary clinic could be of great value for companion animals.

The existing distance between human and veterinary oncology is also evident in the treatment of mammary carcinoma. A plethora of anti-HER2 agents are used or are under investigation to treat this human neoplasm; on the contrary, published related research remains at the *in vitro* phase for canine and feline mammary carcinoma. On a positive note, this opens up avenues for the translation of currently successful and HER2-targeting agents from the human to the veterinary clinic, or even of more affordable generics when available. It is striking to see the lack of research regarding targeting of PDGFRs in canine melanoma, HER2 in canine invasive TCC, CXCR4 in mammary carcinoma and IGF-1R in canine osteosarcoma, all highly expressed molecular targets whose targeting is being evaluated in the human clinic for the respective malignancies. The results of the ongoing human trials, when encouraging, will hopefully spur research in the veterinary setting. In the opposite direction, from the veterinary to the human setting, while targeting of folate receptors has shown great promise in the treatment of dogs with invasive TCC, no reports have been found in human TCC patients and all seems to indicate that also these patients could benefit from the approach.

It is well known that tumor heterogeneity plays a crucial role in the development of drug resistance and patients can clearly benefit from multimodal approaches affecting multiple protumoral pathways. Drawing conclusions from the reviewed data in humans and companion animals, combinations should eventually be considered for the treatment of OSA (including EGFR or PDGFR targeting), HNSCC (with c-Kit inhibition), and mammary carcinoma (using multi-TKIs). As evidenced by this review, the majority of targeted therapies investigated in the veterinary clinic consist of monoclonal antibodies and TKIs. Nowadays, an increasing number of other targeting agents (such as inhibitors of the proteasome and heat shock proteins) are also being assessed, mainly in dogs with hematologic cancers 173. Advances in the human oncology field and the growing interest in comparative oncology will certainly expand the exploration of more and novel targeted therapies in veterinary patients.

The benefits that can arise from an intricate relationship between human and veterinary clinic are nicely exemplified by the successful story of the drug toceranib phosphate, the first agent approved for anticancer targeted therapy in dogs. The evaluation of toceranib phosphate in dogs with different spontaneous cancers showed the first evidence for the use of an oral TKI as cancer treatment. These findings helped the development of its structural analogue, sunitinib, which is currently approved as a treatment for several human cancers 9. Interestingly, the analogue intended for human use received approval in 2006, 3 years before toceranib phosphate was approved in the veterinary clinic, which evidences again the reasonably greater interest in human medicine. Overall, this is an example of successful implementation of comparative oncology in the field of targeted therapy, beneficial for both human and veterinary patients. Within this field, other multi-TKI are also starting to show this benefit across species. Dasatinib, already approved in the human clinic, is now being evaluated in veterinary patients 137. In the opposite direction, masitinib is currently being evaluated in humans, but was first approved to treat companion animals 138. Another good example of this comparative approach is the case of NHS-IL12, an antibody-cytokine conjugate. This immunotherapy was first evaluated in dogs with melanoma to study its safety, antitumor activity and pharmacological properties, which informed the design of the first-in-human clinical trials 175.

The limited development of targeted therapies in veterinary oncology can be ascribed to several factors: from biological concerns such as the lack of epitope homology between the human and canine/feline target or the hypersensitive reaction to species antibodies, to economic reasons such as a less attractive veterinary oncology market for the industry or the few opportunities for funding 176. Clinical trials in companion animals allow for data to be collected relatively fast in comparison to human studies. Still, completion of a study may take over a year and carries associated costs, particularly regarding large scale drug production and the clinical services, facilities and instrumentation. Another difficulty is the recruitment of cases for a veterinary trial, which will be dependent on the incidence of the particular cancer and the relatively early diagnosis to allow for treatment benefit. In this respect, a close collaboration with regional veterinary clinics can definitely be of value. One should not forget that treatment-related toxicities are less acceptable in veterinary medicine, a reason why many successful medicines in the human clinic are not being evaluated in veterinary patients. Opportunities may arise here for targeted therapies, which are known to be tumor-selective to some extent, and thus provide a safer setting. To minimize adverse effects, not only is important to verify the presence of the target at the tumor, but also overall target expression and the interactions of the drug with the species target. Although not common, surface expression pattern between species can differ significantly. This led, for instance, to the TGN1412 catastrophe in 2006 177. In this respect, theranostic strategies that combine targeted detection before therapy seem a safe approach. Also important is the realization that similar expression pattern does not necessarily translate to identical biological behaviour across species. While most studies in the field rely on immunohistochemistry and PCR techniques to assess expression of a handful of targets, only recently more sophisticated techniques are starting to be introduced that allow for unbiased cross-species analysis, such as wide-genome characterization of the cancer 178 and RNA-sequencing of subpopulations of stroma and neoplastic cells 14, 179. Such unbiased analysis enables to investigate similarities between species and to identify new therapeutic targets.

Altogether, when relevant and feasible, breaking the current linearity of drug development programs (mainly from rodents to humans) by incorporating veterinary patients can guide the successful implementation of therapeutics in humans, or aid in identifying failure earlier.

## Conclusion

The use of comparative oncology for translational research of targeted therapies is of benefit for both human and veterinary patients. While improving the translational success rate of these therapies from preclinical to clinical studies, dogs and cats get access to otherwise inaccessible novel therapies. Equally important, targeting therapeutic agents approved for human use can be a potential treatment for dogs and cats with tumors expressing the molecular target in question. Clearly, researchers in the field of veterinary and human oncology could help each other and, more importantly, their patients by working closely together.

## Figures and Tables

**Table 1:** Overview of the translational cancer models included in this review, i.e. human and canine/feline counterpart, available targeted therapies and selected reviews describing similarities

Cancer	% of all cancers	Approved targeted therapy (target)	Selected review
Human head and neck squamous cell carcinoma	6% 1	Cetuximab (EGFR)	13, 14
Canine and feline oral squamous cell carcinoma	8 % of all feline cancers 15 2% of all canine cancers 16	-	
Human invasive transitional cell carcinoma (TCC)	3% are bladder cancer, of which 20% are invasive TCC 1	Erdafitinib (FGFR)	17
Canine invasive TCC	2% are bladder cancer, of which > 90% are invasive TCC 17	-	
Human mammary carcinoma	12% (26% in women) 1	Trastuzumab (HER2), Bevacizumab (VEGF), others	10, 18
Canine and feline mammary carcinoma	25% in female dogs 18; 17% in female cats 19	-	
Human osteosarcoma	3.5% of all childhood cancers < 1% in adults 20	Sorafenib (multiple kinases)	21
Canine osteosarcoma	2% (especially large size breeds) 22	-	
Human melanoma (non-UV induced)	6% are melanoma, of which 15 % are non-UV induced 23	Vemurafenib (BRAF), Trametinib (MEK), others	24
Canine melanoma	7% are melanoma, of which > 95% are non-UV induced 25	-	

EGFR: epidermal growth factor receptor; FGFR: fibroblast growth factor receptor; HER2: human epidermal growth factor receptor 2; VEGF: vascular endothelial growth factor; BRAF: B-Raf proto-oncogene; MEK: mitogen-activated protein kinase.

**Table 2:** Overview of the membrane targets included in this review and their relevance in oncology

Membrane target	Ligands/activation	Protein type	Role	Approved therapies (indication) *or in trials*	Relevance in oncology
EGFR (ErbB1, HER1)	EGF, TGFα, others	Tyrosine kinase receptor	Cell proliferation; migration; angiogenesis; survival	Cetuximab (colorectal cancer), gefitinib (lung cancer), others	EGFR mutations affecting its expression or activity are common drivers of molecular pathogenesis in many cancers 28
HER2 (ErbB2)	Dimerization with other ErbB family receptors			Trastuzumab and lapatinib (breast and gastric cancer), others	HER2 is an established therapeutic target in a subset of human breast cancers first described in the late 1980s 29
PDGFRα/β	PDGF isoforms			Oralatumab (soft tissue sarcoma), imatinib (chronic myeloid leukaemia), others	PDGFRs are among the most commonly inhibited kinases by FDA-approved TKIs to treat cancer 30
c-Met (HGFR, Met)	HGF			Cabozantinib (renal cell carcinoma) and crizotinib (lung cancer)	c-Met deregulation correlates with poor prognosis in many human cancers 31
IGF-1R	IGF-1, IGF-2			*Xentuzumab (lung, prostate and breast cancer)*	When deregulated, IGF-1R plays a role in the pathogenesis of cancer, although it is usually not considered as the main driver 32
IGF-2R	IGF-2, mannose-6-phosphate-bearing molecules	Receptor lacking tyrosine kinase activity	Scavenger for circulating IGF-2	-	Mutations in the *M6P/IGF2R* gene are frequently found in malignant tumors such as melanoma and mammary carcinoma 33
FRα	Folate	GPI-anchored glycoprotein	Regulate uptake of folate (vitamin B9)	*Farletuzumab (ovarian cancer), others*	Overexpression of FRα has been associated with several cancers, whereas its expression in normal tissues is limited 34
EP2	PGE2	G protein-coupled receptor	Angiogenesis, metastasis, treatment resistance	*TPST-1495 (colorectal cancer)*	EP2 is abnormally expressed in many cancers, including prostate, bladder and breast cancers 35
MCTs	Lactate, pyruvate ketone bodies, others	Proton-linked channel	Transmembrane transport of monocarboxylate metabolites	*AZD3965 (lymphoma)*	Due to the high glycolytic activity of tumor cells, MCT1 and MCT4 are highly expressed by aggressive and metastasizing tumors 36
CD146 (MCAM)	Laminin 411, VEGFR2, others	Immunoglobulin superfamily receptor	Adhesion molecule and cell survival	-	In human melanomas, CD146 is largely involved in tumor progression 37
CXCR4 (CD184)	CXC12	G protein-coupled receptor	Cell proliferation and survival	*Ulocuplumab (multiple myeloma)*	CXCR4 expression is low or absent in many healthy tissues, but it is expressed in over 20 types of cancer 38

EGFR: epidermal growth factor receptor; TGFα: transforming growth factor alpha; HER2: human epidermal growth factor receptor 2; PDGFR-α/β: platelet derived growth factor receptor α/β; c-Met: tyrosine-protein kinase Met; HGFR: hepatocyte growth factor receptor; TKI: tyrosine kinase inhibitor; IGF-1/2R: insulin-like growth factor 1/2 receptor; FRα: folate receptor α; GPI: glycosylphosphatidylinositol; EP2: prostaglandin E2 receptor 2; PGE2: prostaglandin E2; MCT1/4: monocarboxylate transporter 1/4; CD146: cluster of differentiation 146; MCAM: melanoma cell adhesion molecule; VEGFR: vascular endothelial growth factor receptor; CXCR4: C-X-C chemokine receptor type 4; CXC12: C-X-C chemokine 12.

**Table 3:** Overview of the use of toceranib phosphate, dasatinib and masitinib mesylate in veterinary clinical studies and *in vitro*

Cancer	Toceranib phosphate	Dasatinib	Masitinib mesylate	Ref.
HNSCC	Dogs & cats		Canine & feline cells	134, 135, 139
TCC	Dogs			140
OSA	Dogs	Dogs		135, 141, 142
MC	Dogs			143, 144
MM	Dogs		Dogs	144, 145

HNSCC: head and neck squamous cell carcinoma; TCC: transitional cell carcinoma; OSA: osteosarcoma; MC: mammary carcinoma; MM: malignant melanoma.

**Table 4:** Overview of the molecular targets and targeted therapy drugs for canine and feline cancers discussed in this review

Molecular target	Homology dog (%)*	Homology cat (%)*	Cancer	Targeted agent	Tested in	Ref.
EGFR	91.5	91.7	HNSCC	Gefitinib	Feline cells	44, 45
				siRNA	Feline cells	44
			TCC	EGF‐anthrax toxin	Dogs	49
				Can225IgG-IRDye700DX	Canine xenograft mouse	50
			OSA	Gefitinib	Canine cells	54
				Erlotinib	Canine cells	55
				Erbstatin	Canine cells	56
			MC	Cetuximab	Canine cells	61
				Gefitinib	Canine/feline cells	62
				Can225IgG	Canine cells	63
				siRNA	Canine cells	62
			MM	-	-	67
HER2	93.7	92.9	TCC	-	-	70-72
			OSA	-	-	77
			MC	Trastuzumab	Canine cells	61
				AG825	Canine/feline cells	62
				siRNA	Canine/feline cells	62
			MM	-	-	67
PDGFRα/β	95.9/90.2	95.4/89.8	OSA	-	-	91
			MM	-	-	95
c-Met	89.9	90.0	OSA	shRNA	Canine cells	98
				PHA‐665752	Canine cells	98
				PF2362376	Canine cells	101
IGF-1R	98.2	98.1	OSA	-	-	103, 104
			MM	-	-	109-111
IGF-2R	85.6	85.7	OSA	-	-	116
FRα	80.5	75.7	TCC	EC0905	Dogs	118
				EC0531	Dogs	119
EP2	89.0	92.1	TCC	-	-	72
MCT1/4	88.6/87.6	88.4/88.0	HNSCC	MD-1	Feline xenograft mouse	124
CD146	81.9	83.6	MM	-	-	127, 128
CXCR4	95.8	94.9	MC	-	-	130-132

*The protein sequences of the human, canine and feline molecular targets were found in the Protein NCBI database and aligned with Protein BLAST having human as the reference sequence. EGFR: epidermal growth factor receptor; HER2: human epidermal growth factor receptor 2; PDGFR-α/β: platelet derived growth factor receptor α/β; c-Met: tyrosine-protein kinase Met; IGF-1/2R: insulin-like growth factor 1/2 receptor; FRα: folate receptor α; EP2: prostaglandin E2 receptor 2; MCT1/4: monocarboxylate transporter 1/4; CD146: cluster of differentiation 146; CXCR4: C-X-C chemokine receptor type 4; HNSCC: head and neck squamous cell carcinoma; TCC: transitional cell carcinoma; OSA: osteosarcoma; MC: mammary carcinoma; MM: malignant melanoma.

## Abbreviations

BRAF: B-Raf proto-oncogene
CMC: canine mammary carcinoma
COX2: cyclooxygenase 2
CXCR4: C-X-C chemokine receptor type 4
EGFR: epidermal growth factor receptor
EP2: prostaglandin E2 receptor 2
FMC: feline mammary carcinoma
FGFR: fibroblast growth factor receptor
FR: folate receptor
HER2: human epidermal growth factor receptor 2
HNSCC: head and neck squamous cell carcinoma
IGF: insulin-like growth factor
IGF-1/2R: insulin-like growth factor 1/2 receptor
MCT: monocarboxylate transporter
MEK: mitogen-activated protein kinase
NSAID: non-steroidal anti-inflammatory drugs
OSA: osteosarcoma
OSCC: oral squamous cell carcinoma
PDGFR-α/β: platelet derived growth factor receptor α/β
TCC: transitional cell carcinoma
TKI: tyrosine kinase inhibitor
VEGF: vascular endothelial growth factor
VEGFR: vascular endothelial growth factor receptor
